# Trousseau's syndrome in diffuse large B‐cell lymphoma

**DOI:** 10.1002/jha2.837

**Published:** 2023-12-15

**Authors:** Wataru Kitamura, Yumiko Sato, Shoichi Kuyama

**Affiliations:** ^1^ Department of Hematology National Hospital Organization Iwakuni Clinical Center Iwakuni Japan; ^2^ Department of Hematology, Oncology and Respiratory Medicine Okayama University Graduate School of Medicine, Dentistry and Pharmaceutical Sciences Kita‐ku Japan; ^3^ Department of Pathology National Hospital Organization Iwakuni Clinical Center Iwakuni Japan; ^4^ Department of Respiratory Medicine National Hospital Organization Iwakuni Clinical Center Iwakuni Japan

**Keywords:** diffuse large B‐cell lymphoma, multiple cerebral infarctions, Trousseau's syndrome

1

A 62‐year‐old Japanese female was referred to our institution with complaints of headache and vertigo. Her medical history included atopic dermatitis. Brain diffusion‐weighted magnetic resonance imaging (MRI) revealed multiple cerebral infarctions spanning various vascular territories (Figure 1A,B). Cervical magnetic resonance angiography showed no obvious carotid artery stenosis. Contrast‐enhanced T1‐weighed brain MRI did not demonstrate any lesions with enhancement (Figure 1C,D). Laboratory studies indicated a normal complete blood count but showed elevated levels of lactate dehydrogenase (415 IU/L; reference range, 124–222 IU/L) and soluble interleukin‐2 receptor (1163 IU/mL; reference range, 122–496 IU/mL). Antithrombin, protein C, and protein S levels were within the normal range, and various autoantibodies such as anti‐Smith antibodies, anti‐double Stranded DNA antibodies, lupus anticoagulant, antiphospholipid antibodies, and antineutrophil cytoplasmic antibodies were not detected. There were no abnormalities found in transthoracic echocardiography or 24‐h electrocardiographic monitoring. Cerebrospinal fluid cytology revealed no malignant tumor cells. Positron emission tomography‐computed tomography demonstrated increased fluorodeoxyglucose uptake in a mass located from the pelvis to the left inguinal region (Figure 1E, red arrowheads). Biopsy specimens of the left inguinal tumor, stained with hematoxylin and eosin, demonstrated diffuse infiltration of abnormal cells with large and irregular nuclei (Figure 1F). These abnormal cells were CD3−, CD5−, CD10−, CD20+, BCL6+, and MUM1− on immunohistochemistry (Figure 1G). Immunoglobulin heavy chain (*IgH*)*/BCL2* fusion signal and *BCL6* and *MYC* split signal were not detected by fluorescence in situ hybridization analysis. Based on these results, we diagnosed the patient with diffuse large B‐cell lymphoma (Hans’ algorithm: [1] germinal center B cell type) complicated by Trousseau syndrome. The patient's symptoms improved after receiving rituximab in combination with chemotherapy.

**FIGURE 1:**
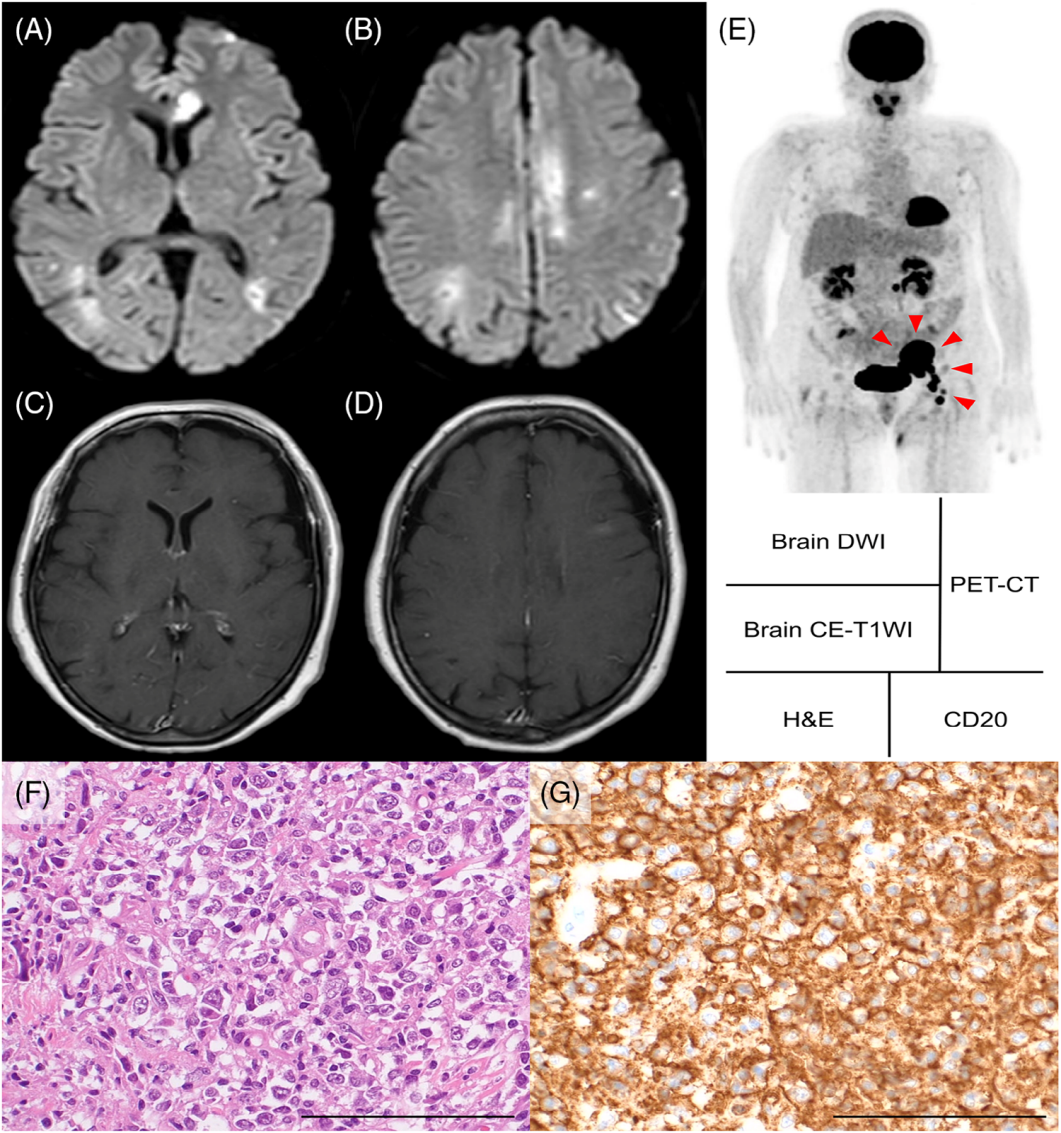
(A, B) Brain diffusion‐weighted magnetic resonance imaging (MRI) shows multiple cerebral infarctions. (C, D) Contrast‐enhanced T1‐weighed brain MRI shows no lesions with contrast effect. (E) Positron emission tomography‐computed tomography (PET‐CT) reveals the increased uptake of fluorodeoxyglucose in a mass found from the pelvis to left inguinal (red arrowheads). (F) Hematoxylin and eosin staining (H&E) shows large abnormal lymphocytes (×200, scale bar: 100 μm). (G) These abnormal lymphocytes are positive for CD20 on immunohistochemistry staining (×200, scale bar: 100 μm). DWI, diffusion‐weighted image; CE‐T1WI, contrast‐enhanced T1‐weighed image

Trousseau syndrome is initially defined as “a hypercoagulable state associated with malignant tumor and resulting in migratory venous thromboembolism,” but today the clinical presentation includes deep vein thrombosis, pulmonary embolism, chronic disseminated intravascular coagulation with nonbacterial thrombotic endocarditis (NBTE), and arterial thrombosis [2]. Several reports have suggested that cardiogenic embolism caused by NBTE was more important than local thrombus formation caused by hypercoagulability as a mechanism of stroke in patients with cancer [3, 4, 5]. It is known that adenocarcinoma is often the underlying disease of Trousseau syndrome [2]. On the other hand, in the context of hematopoietic tumor, intravascular large B‐cell lymphoma can sometimes present with cerebral infarct‐like lesions due to direct occlusion of blood vessels by the lymphoma cells themselves [6]. However, it is extremely rare for other lymphomas to be the underlying cause of true multiple cerebral infarctions [7]. Our case highlights the importance of considering lymphoma as a potential cause, in addition to solid tumors, when physicians encounter patients with multiple cerebral infarctions of unknown origin.

## AUTHOR CONTRIBUTIONS

W.K. collected clinical, radiological, and histological data and wrote the original draft. Y.S. performed a histological diagnosis. S.K. performed supervision, review, and editing. All authors approved the final manuscript.

## CONFLICT OF INTEREST STATEMENT

The authors declare that they have no conflict of interest.

## FUNDING INFORMATION

No funding was received for this work.

## ETHICS APPROVAL STATEMENT

The authors have confirmed ethical approval statement is not needed for this submission.

## PATIENT CONSENT STATEMENT

A written informed consent was obtained from the patient for the publication.

## PERMISSION TO REPRODUCE MATERIAL FROM OTHER SOURCES

N/A.

## CLINICAL TRIAL REGISTRATION

The authors have confirmed clinical trial registration is not needed for this submission.

## Data Availability

The data in this study are available from the corresponding author upon reasonable request.
